# Transcriptional profiling of UlaR-regulated genes in *Streptococcus pneumoniae*

**DOI:** 10.1016/j.gdata.2015.02.004

**Published:** 2015-03-07

**Authors:** Sulman Shafeeq, Muhammad Afzal, Birgitta Henriques-Normark, Oscar P. Kuipers

**Affiliations:** a Department of Microbiology, Tumor and Cell Biology, Karolinska Institutet, Nobels väg 16, 17177 Stockholm, Sweden; b Department of Molecular Genetics, Groningen Biomolecular Sciences and Biotechnology Institute, University of Groningen, Nijenborgh 7, 9747 AG Groningen, The Netherlands; c Department of Bioinformatics and Biotechnology, G C University, Faisalabad, Pakistan

**Keywords:** Ascorbic acid *Streptococcus pneumoniae*, UlaR, Microarray, Bacteria

## Abstract

The transcriptional regulator UlaR belongs to the family of PRD-containing transcriptional regulators, which are mostly involved in the regulation of carbohydrate metabolism. The role of the transcriptional regulator UlaR in *Streptococcus pneumoniae* has recently been described [1]. Here, we report detailed genome-wide transcriptional profiling of UlaR-regulated genes in *S. pneumoniae* D39 and its ∆*ulaR* derivative, either in the presence of 10 mM ascorbic acid in M17 medium using microarray analysis. 10 mM concentration of ascorbic acid was supplemented to the M17 medium because our *lacZ*-fusion studies indicated that UlaR acts as a transcriptional activator of its targets in the presence of ascorbic acid and the expression of the *ula* operon was maximal at a 10 mM ascorbic acid concentration [1]. All transcriptional profiling data of UlaR-regulated genes was deposited to Gene Expression Omnibus (GEO) database under accession number GSE61649.

**Table:** 

Specifications	
Organism/cell line/tissue	*Streptococcus pneumoniae* D39 strain
Sex	N/A
Sequencer or array type	Oligo-based DNA microarray
Data format	Raw and processed
Experimental factors	D39 ∆*ulaR* versus D39 wild-type
Experimental features	Microarray comparison was preformed to identify genes differentially expressed in D39 ∆*ulaR* compared to D39 wild-type in M17 medium with 10 mM ascorbic acid
Consent	N/A
Sample source location	Groningen, The Netherlands

## Direct link to deposited data

Microarray data is accessible under the following link: http://www.ncbi.nlm.nih.gov/geo/query/acc.cgi?acc=GSE61649

## Experimental design, materials and methods

### Growth conditions

The list of strains used for transcriptome analysis is mentioned in Table 1. To analyze the effect of the *ulaR* deletion on the transcriptome of *S. pneumoniae*, the D39 wild-type strain and its mutant (D39 ∆*ulaR*) strain were grown at 37 °C in replicates (50 ml each) in AM17 (10 mM Ascorbic acid + M17) medium and harvested at their respective mid-exponential growth phase by centrifugation at 10,000 rpm for 1 min at 4 °C. Following centrifugation, the supernatant was carefully removed and the cell pellet was immediately frozen in liquid nitrogen. Cell pellets were further stored at − 80 °C. All other procedures regarding the DNA microarray experiment were performed as described previously [2].

### RNA extraction

RNA extraction was performed as described before [3]. In short, frozen cell pellets were resuspended in 400 μl TE (DEPC) and transferred to the screw-capped tubes containing 0.5 g glass beads, 50 μl 10% SDS and 500 μl phenol:chloroform:isoamyl alcohol (25:24:1). The cells were lysed by using a bead beater. The lysed samples were then centrifuged at 10,000 rpm for 10 min (4 °C). The upper phase was transferred to fresh tubes containing 500 μl chloroform:IAA (24:1) and centrifuged for 5 min at 10,000 rpm (4 °C). The 500 μl of upper phase was again transferred to fresh tubes containing 2 × volumes (1 ml) of lysis/binding buffer provided with high pure RNA isolation kit (Roche international). All other steps including the DNaseI were performed according to the manual provided with high pure RNA isolation kit. The concentration of RNA was checked on a spectrophotometer and quality of RNA was checked using Agilent RNA analysis kit (Agilent technologies).

### cDNA preparation and labeling

RNA (10–15 μg) was mixed with 2 μl random nonamers (1.6 μg/μl) and nuclease free water was added if necessary to keep the final volume of the annealing mixture to 18 μl. The annealing mixture was kept at 70 °C for 5 min. After 5 min of incubation at 70 °C, annealing mixtures were cooled at room temperature and 12 μl of master mix (consisting of 6 μl 5 × first strand buffer, 3 μl 0.1 M DDT, 1.2 μl 25 × AA-dUTP / nucleotide mix and 1.8 μl Superscript III reverse transcriptase) was added to the annealing mix and the reaction mix was incubated at 42 °C for 2–16 h. Following the incubation, the mRNA from the reaction mixture was degraded by adding 3 μl of 2.5 M NaOH and placing the reactions at 37 °C for 15 min. To neutralize the NaOH in reactions, 15 μl of 2 M HEPES free acid was added. The cDNA mixture was purified by using PCR clean-up columns and following the manufacturer's protocol. DyLight-550 and DyLight-650 were used to label the cDNA samples in dye-swap manner.

### Hybridization and washing

The labeled cDNA was used for hybridization with not more than 30% difference in the cDNA concentration. 0.5 pmol/μl was taken as the minimum concentration of DyLight550 or DyLight650 in a total eluted volume of 50 μl. The cDNA samples were mixed accordingly and hybridized for around 16 h at 45 °C in Ambion Slidehyb #1 hybridization buffer (Ambion Europe) on in-house produced super-amine glass slides (Array-It), comprising three technical replicates of each amplicons denoting 2087 ORFs of *S. pneumoniae* TIGR4 [4] and 184 ORFs specific for *S. pneumoniae* R6 [5]. After hybridization, slides were washed using appropriate washing buffers.

### Microarray data analysis

Microarray slides were scanned and pre-analyzed using “GenePix Pro 6” software as described previously [6]. Raw data files were also deposited on GEO under the accession number GSE61649. Further normalization and processing of the data were performed with in-house developed *Microprep* software package as described before [7]. Statistical analyses were performed as described previously [8]. Independent biological replicates for DNA microarray data were used, which were dye-swapped. The measurements of at least 5 spots were used to calculate the expression ratios of the D39 ∆*ulaR* strain over the D39 wild-type strain. CyberT implementation of a variant of *t-test* (http://bioinformatics.biol.rug.nl/cybert/index.shtml) was performed and false discovery rates (FDRs) were calculated as described [7]. For differentially expressed genes, p < 0.001 and FDR < 0.05 were taken as standard. Further computational analysis on the data for the regulatory network prediction and data mining was done using different software packages [9], [10], [11], [12]. Microarray data have been submitted to GEO under the accession number GSE61649. For the identification of differentially expressed genes a Bayesian p-value of < 0.001 and a fold change cut-off twofold were applied.

## Discussion

Here, we investigated the impact of an *ulaR* mutation on the transcriptional profile of *S. pneumoniae* D39. Transcriptome comparison of the D39 ∆*ulaR* with the D39 wild-type grown in AM17 (10 mM Ascorbic acid + M17) revealed the specific effect on the gene expression of *S. pneumoniae*. 12 genes were downregulated and 9 genes were upregulated in the D39 ∆*ulaR* strain compared to the D39 wild-type in the presence of ascorbic acid (Table 2). The *ula* operon was highly downregulated in the D39 ∆*ulaR* strain confirming the role of UlaR as a transcriptional activator of the *ula* operon in the presence of ascorbic acid. This is further confirmed by β-galactosidase assays and promoter truncation experiments [1], [13]. The expression of some other genes was also altered in our transcriptome analysis, but further investigations are required to clear the role of transcriptional regulator UlaR in the regulation of these genes.

## Conflict of interest

The authors have no conflicts of interest.

## Figures and Tables

**Table 1:** List of pneumococcal strains used in microarray study.

Strain/plasmid	Description	Source
D39	Serotype 2 strain. *cps 2*	Laboratory of P. Hermans
Δ*ulaR*	D39 Δ*ulaR*; Spec^R^	[1]

**Table 2:** Summary of transcriptome comparison of *S. pneumoniae* strain D39 Δ*ulaR* and D39 wild-type grown in AM17 (10 mM Ascorbic acid + M17).

D39 tag^a^	Function^b^	Ratio^c^	p-Values
*spd_0063*	Beta-N-acetylhexosaminidase, StrH	− 2.3	6.58E-07
*spd-0450*	Type I restriction-modification system, S subunit, putative	− 4.0	4.98E-08
*spd-0452*	Integrase/recombinase, phage integrase family protein	3.7	3.40E-11
*spd-0608*	Orotidine 5′-phosphate decarboxylase, PyrF	2.1	1.50E-03
*spd-1046*	6-phospho-beta-galactosidase, LacG	2.4	1.39E-08
*spd-1047*	PTS system, lactose-specific IIBC components, LacE	2.4	2.12E-07
*spd-1107*	Guanosine monophosphate reductase, GuaC	2.4	1.19E-07
*spd-1131*	Carbamoyl-phosphate synthase, large subunit, CarB	2.3	1.62E-07
*spd-1302*	Oxidoreductase, putative	− 2.0	2.03E-03
*spd-1324*	IS630-Spn1, transposase Orf2	2.4	9.43E-03
*spd-1413*	ATP-dependent RNA helicase, putative	2.3	4.52E-07
*spd_1839*	Transketolase, Tkt	− 2.3	9.58E-09
*spd_1840*	l-ascorbate 6-phosphate lactonase, UlaG	− 26.1	4.16E-09
*spd_1841*	BglG family transcriptional regulator, UlaR	− 7.0	4.42E-08
*spd_1842*	l-ribulose-5-phosphate 4-epimerase, UlaF/AraD	− 10.1	1.81E-07
*spd_1843*	l-xylulose 5-phosphate 3-epimerase, UlaE	− 19.1	1.07E-12
*spd_1844*	3-keto-l-gulonate-6-phosphate decarboxylase, UlaD	− 25.8	4.50E-13
*spd_1845*	Ascorbate-specific PTS system, IIA component, UlaC	− 12.4	8.74E-09
*spd_1846*	Ascorbate-specific PTS system, IIB component, UlaB	− 24.7	7.72E-10
*spd_1847*	Ascorbate-specific PTS system, IIC component, UlaA	− 9.5	1.75E-08
*spd_1913*	Phosphate ABC transporter, ATP-binding protein, PstB	2.1	2.30E-04

a Gene numbers refer to D39 locus tags.

b D39 annotation/TIGR4 annotation [14].

c Ratio represents the fold decrease in the expression of genes in D39 Δ*ulaR* compared to the D39 wild-type.
